# The association of medical mistrust, clinical trial knowledge, and perceived clinical trial risk with willingness to participate in health research among historically marginalized individuals living in New York City

**DOI:** 10.1007/s44250-025-00336-1

**Published:** 2026-01-07

**Authors:** Isabel Inez Curro, Laura Wyatt, Victoria Foster, Yousra Yusuf, Sonia Sifuentes, Perla Chebli, Julie A. Kranick, Simona C. Kwon, Chau Trinh-Shevrin, Madison N. LeCroy

**Affiliations:** 1 Department of Epidemiology & Biostatistics, CUNY Graduate School of Public Health and Health Policy, City University of New York, New York, NY USA; 2 Department of Population Health, Section for Health Equity, NYU Grossman School of Medicine, New York, 10016 NY USA

**Keywords:** Research participation, Medical mistrust, Health research knowledge, Perceived research risk, Racial and ethnic groups, Health disparities

## Abstract

Medical mistrust, clinical trial knowledge, and clinical trial risk impact research participation, yet are rarely studied among racial and ethnic groups. Data were from a cross-sectional survey (*n* = 1,794). Multinomial logistic regression models examined associations of medical mistrust, clinical trial knowledge, and clinical trial risk with willingness to participate in health research (Yes, No, Unsure) among Chinese, Korean, South Asian, Haitian, North American Latiné, South American Latiné, and Southwest Asian and North African (SWANA) NYC residents with one model per group. Overall, 46.0% of participants reported willingness to participate, ranging from 35.8% (Chinese participants) to 58.7% (South Asian participants). Increased mistrust was associated with less willingness among Chinese (OR: 1.06, 95%CI: 1.00, 1.12) and South American Latiné (OR: 1.15, 95%CI: 1.02, 1.30) participants; more willingness among Haitian participants (OR: 0.87, 95%CI: 0.81, 0.94); more uncertainty among Korean (OR: 1.13, 95%CI: 1.05, 1.22), South Asian (OR: 1.06 95%CI: 1.01, 1.12), and North American Latiné (OR: 1.18, 95%CI: 1.10, 1.28) participants; and less uncertainty among Haitian (OR: 0.91, 95%CI: 0.84, 0.99) and SWANA (OR: 0.91, 95%CI:0.86, 0.97) participants. Knowledge was associated with more willingness for Haitian participants (OR: 2.77, 95%CI: 1.15, 6.65), less willingness for Chinese participants (OR: 0.55, 95%CI: 0.34, 0.88), and more uncertainty among South Asian (OR: 2.09, 95%CI: 1.07, 4.07) and SWANA (OR: 2.71, 95%CI: 1.21, 6.03) participants. Some risk and more willingness were linked for South American Latiné participants (OR: 0.13, 95%CI: 0.02, 0.82). Associations varied by group. Studying multiple racial and ethnic groups advances equitable research representation.

## Introduction

### Lack of diverse participation in medical research

The 1993 National Institutes of Health Revitalization Act requires ethnically and racially diverse individuals to be included in health research, aiming to reduce health disparities [1]. Yet, from 1993 to 2018, 96% of United States (U.S.) randomized controlled trial (RCT) participants were non-Hispanic White [2]. Alaskan Native/Native American, Asian, Black/African American, Latiné (i.e., Spanish-speakers, those descended from Spanish-speakers, and those from or descended from Latin America), and Native Hawaiian/Pacific Islander individuals were underrepresented in RCTs by ≥ 450% compared to their respective share of the U.S. population (Alaskan Native/Native American: 0.4% vs. 2.9%; Black/African American: 2.2% vs. 12.4%; Latiné: 1.2% vs. 18.9%; Asian: 1.0% vs. 6.0%; Native Hawaiian/Pacific Islander 0.04% vs. 0.2%) [2, 3]. Though no data was reported for Middle Eastern/North African or Multiracial/Multiethnic individuals, these groups are also included in the U.S. Office of Management and Budget’s minimum reporting categories [4]. Similar underrepresentation exists among limited English proficiency (LEP) individuals [5–7], a quickly growing group which makes up 9% of the U.S. population [5]. According to the National Academies, this underrepresentation worsens health disparities, limits access to effective treatments, and could cost hundreds of billions of dollars in life expectancy, disability-free years, and labor force participation over the next three decades [8]. 

Despite these serious concerns, the 1993 Revitalization Act remains poorly enforced, with minimal gains in racially and ethnically diverse study populations. This persistent underrepresentation is likely due to several remediable factors, including the dearth of education, training, motivation, and incentives for researchers to design and implement diverse studies and to recruit and retain diverse participants. Additional factors include the underrepresentation of racially and ethnically diverse research and medical professionals, which likely contributes to the lack of partnerships between academic medical centers and underrepresented communities [9, 10]. Yet, findings from this largely non-inclusive research continue to support new medication approvals and validate evidence-based medical practices.

### Medical mistrust, health research knowledge, and perceived health research risk

The U.S. medical system’s historical mistreatment of racial and ethnic groups contributes to ongoing health inequities [11]. Egregious abuses, such as conducting violent experiments on enslaved Black people, the Tuskegee Syphilis Study—where researchers did not obtain informed consent from the 600 African American participants and withheld diagnosis information and treatment from the 399 participants with syphilis—and forced sterilizations of Native, Latiné, and Black individuals, have fueled medical mistrust: the belief that medical institutions may act against patients’ best interests [12]. Mistrust of both the U.S. healthcare and medical research institutions is common in the U.S [12, 13].

Experiencing systemic racism and interacting with biased institutions are “basic causes” of medical mistrust [12]. In the U.S. medical system, biases may be experienced as discrimination by one’s healthcare provider or inadequate culturally and linguistically appropriate health communication [12, 14]. Mistrust hinders medical research participation, especially among racially and ethnically diverse and LEP U.S. residents [13, 15, 16]. 

Health research knowledge and perceived risk associated with participation are also linked to participation willingness. George et al.’s 2014 systematic review on factors impacting research participation found that, besides medical mistrust, lack of health research knowledge was the only barrier shared across all included groups—African American, Latiné, Asian American, and Pacific Islander [11]—highlighting both the importance of knowledge to research participation and the heterogeneity of barriers to participation across differing groups. Among African American, Latiné, and Asian American participants, fear of adverse effects was another barrier to participation and perceived low risk of research participation was a facilitator [11]. Among LEP individuals within these groups, the common lack of bilingual research staff or translated research materials presented additional barriers to participation.

The impact of medical mistrust, health research knowledge, and perceived participation risk on participation willingness have been explored among some racial and ethnic U.S. groups, including African American and Latiné communities. However, few studies have examined these themes among further disaggregated ethnic, national, or regional subgroups (e.g., Chinese, Mexican). Further, other diverse groups residing in the U.S., including Korean, South Asian, Haitian, Central and South American, and Southwest Asian and North African (SWANA; formerly known as Middle Eastern/North African), are missing from the literature altogether.

### Data disaggregation

Previous studies among Asian Americans have disaggregated data by racial and ethnic groups to show differences within broader aggregated groups [17–20], and the New York City (NYC) Department of Health and Mental Hygiene regularly presents racially and ethnically disaggregated public health reports, including among Black, Latiné, and Asian residents [21–23]. Despite challenges with small sample sizes and higher risk of re-identification, racially and ethnically disaggregated analyses improve validity of research findings by reducing misclassification biases and hidden heterogeneity [24] and enabling more precise targeting of interventions and resource allocation. This can lead to both more efficient resource use and improved health outcomes across racial and ethnic groups. However, the same factors causing persistent non-inclusion of racially and ethnically diverse individuals in research also prevent data collection that supports disaggregation.

This study disaggregates data among one Black American subgroup: Haitian; three Asian American subgroups: Chinese, Korean, and South Asian; and two Latiné subgroups: North American Latiné (Latiné individuals identifying as from or as descendants of individuals from countries in North America, including Mexico, Central America, and the Caribbean) and South American Latiné (Latiné individuals identifying as from or as descendants of individuals from countries in South America).

**Table 1:** Descriptive statistics by racial, ethnic, or regional identity with p-values indicating differences between Chinese, Korean, South Asian. Haitian, North American Latiné, South American Latiné, and SWANA individuals using chi-square and Kruskal-Wallis tests

**Variable**	**Total Sample ** **N (%)**	**All Asian**^a^ **N (%)**	**Chinese ** **N (%)**	**Korean ** **N (%)**	**South Asian**^b^ **N (%)**	**Haitian ** **N (%)**	**All Latine**^c^ **N (%)**	**North American Latiné**^d^ **N (%)**	**South American Latiné**^e^ **N (%)**	**SWANA**^f^ **N(%)**	**P-value**
Total, n (%)	1,794	1066 (59.4%)	537 (29.9%)	243 (13.5%)	286 (15.9%)	149 (8.3%)	315 (17.6%)	220 (12.3%)	95 (5.3%)	264 (14.7%)	
*Willingness to participate in health research*											**<0.0001**
Yes	825 (46.0%)	476 (44.7%)	192 (35.8%)	116 (47.7%)	168 (58.7%)	57 (38.3%)	164 (52.1%)	114 (51.8%)	50 (52.6%)	128 (48.5%)	
No	494 (27.5%)	311 (29.2%)	176 (32.8%)	75 (30.9%)	60 (21.0%)	59 (39.6%)	57 (18.1%)	40 (18.2%)	17 (17.9%)	67 (25.4%)	
Don’t know/Not sure	475 (26.5%)	279 (26.2%)	169 (31.5%)	52 (21.4%)	58 (20.3%)	33 (22.1%)	94 (29.8%)	66 (30.0%)	28 (29.5%)	69 (26.1%)	
*Medical mistrust, mean (SD)*	25.5 (5.4)	24.7 (5.0)	24.3 (4.0)	25.3 (5.3)	24.8 (6.2)	29.0 (6.4)	27.1 (5.1)	26.9 (5.0)	27.7 (5.4)	24.9 (5.5)	**<0.0001**
*Clinical trial knowledge*											**<0.0001**
Very/Somewhat knowledgeable	603 (33.6%)	317 (29.7%)	147 (27.4%)	41 (16.9%)	129 (45.1%)	67 (45.0%)	134 (42.5%)	94 (42.7%)	40 (42.1%)	85 (32.2%)	
Not very/Not at all knowledgeable	1191 (66.4%)	749 (70.3%)	390 (72.6%)	202 (83.1%)	157 (54.9%)	82 (55.0%)	181 (57.5%)	126 (57.3%)	55 (57.9%)	179 (67.8%)	
*Clinical trial risk*											**<0.0001**
A lot of risk	308 (17.2%)	163 (15.3%)	85 (15.8%)	35 (14.4%)	43 (15.0%)	63 (42.3%)	50 (15.9%)	39 (17.7%)	11 (11.6%)	32 (12.1%)	
Some risk	1369 (76.3%)	839 (78.7%)	439 (81.8%)	194 (79.8%)	206 (72.0%)	78 (52.3%)	239 (75.9%)	165 (75.0%)	74 (77.9%)	213 (80.7%)	
No risk at all	117 (6.5%)	64 (6.0%)	13 (2.4%)	14 (5.8%)	37 (12.9%)	8 (5.4%)	26 (8.3%)	16 (7.3%)	10 (10.5%)	19 (7.2%)	
*Age, mean (SD)*	44.3 (17.3)	45.3 (17.6)	49.4 (18.0)	45.3 (17.6)	37.4 (14.0)	37.7 (14.9)	42.0 (15.0)	41.4 (14.9)	43.3 (15.2)	47.1 (18.8)	**<0.0001**
*Sex assigned at birth*											**<0.0001**
Male	682 (38.0%)	431 (40.4%)	201 (37.4%)	106 (43.6%)	124 (43.4%)	55 (36.9%)	78 (24.8%)	54 (24.5%)	24 (25.3%)	118 (44.7%)	
Female	1112 (62.0%)	635 (59.6%)	336 (62.6%)	137 (56.4%)	162 (56.6%)	94 (63.1%)	237 (75.2%)	166 (75.5%)	71 (74.7%)	146 (55.3%)	
*Education level*											**<0.0001**
High school graduate or less	770 (42.9%)	486 (45.6%)	343 (63.9%)	71 (29.2%)	72 (25.2%)	38 (25.5%)	163 (51.7%)	119 (54.1%)	44 (46.3%)	83 (31.4%)	
More than high school	1,024 (57.1%)	580 (54.4%)	194 (36.1%)	172 (70.8%)	214 (74.8%)	111 (74.5%)	152 (48.3%)	101 (45.9%)	51 (53.7%)	181 (68.6%)	
*Nativity*											**<0.0001**
Born in the United States	375 (20.9%)	186 (17.4%)	82 (15.3%)	33 (13.6%)	71 (24.8%)	32 (21.5%)	108 (34.3%)	88 (40.0%)	20 (21.1%)	49 (18.6%)	
Not born in the United States	1413 (79.1%)	880 (82.6%)	455 (84.7%)	210 (86.4%)	215 (75.2%)	117 (78.5%)	207 (65.7%)	132 (60.0%)	75 (78.9%)	215 (81.4%)	

^a^Includes Chinese, Korean, and South Asian individuals

^b^Includes Asian Indian, Bangladeshi, Nepali, and Pakistani individuals

^c^Includes North American Latiné and South American Latiné individuals

^d^Includes Costa Rican, Cuban, Dominican, Guatemalan, Honduran, Mexican, Nicaraguan, Panamanian, Puerto Rican, and Salvadoran individuals

^e^Includes Bolivian, Brazilian, Chilean, Colombian, Ecuadorian, Paraguayan, Peruvian, and Venezuelan individuals

^f^Southwest Asian and North African; includes Afghan, Algerian, Egyptian, Iranian, Iraqi, Israeli, Jordanian, Lebanese, Moroccan, Palestinian, Saudi, Syrian, and Yemeni individuals

^g^Bold indicates statistical significance at 𝛼=0.05

### Importance of distinguishing between North American and South American Latiné

Latiné communities have a long history in NYC, with individuals from North versus South America having distinct migration histories. Puerto Rican and Dominican communities comprised much of the first waves of Latiné immigration to NYC starting in the mid-1800’s and picking up again in the 1930’s-1950’s [20]. These two groups shared many of the same reasons for immigrating, including family reunification, political factors, and economic hardships [26–28]. Mexican individuals followed soon after, and these three groups are the three most populus Latiné groups in NYC [29]. Later, in the mid-1980’s, large waves of immigration from Central America to the U.S. began [30]. Since then, the Central American immigrant population in the U.S. has grown tenfold, with individuals from El Salvador, Guatemala, and Honduras making up the majority of Central American migrating communities [31]. Now, NYC is home to the second highest concentration of Central American immigrants across all metropolitan areas in the U.S [31].

NYC also has the largest South American immigrant population of all U.S. cities, yet the migration history is distinct from that of Mexico and Caribbean and Central American countries [32]. The Cold War era saw the first large waves of immigration into the U.S. from South American countries due to armed conflict and political and economic instability [32]. And despite the overall growth of the South American Latiné immigrant population, there has been a steady decline in South American immigration over the past 20 years [32], perhaps due to increased intracontinental immigration within South America and intercontinental immigration from South America to Spain [33]. Given these differences in migration histories and their potential impact on health and trust, we consider South American Latiné individuals as distinct from individuals from Mexico, the Caribbean, and Central America in the present analysis. We have grouped Latiné individuals from Mexico, the Caribbean, and Central America together as North American Latiné to reflect similar common causes for migration—including a dearth of job opportunities and social and gender-based violence—and that Mexico has historically been the main entry point into the U.S. for individuals from Central America [33]. 

### Present study

Using data from a community needs assessment designed to understand health-related challenges, needs, and resources of diverse and minoritized NYC populations, we examined whether medical mistrust or perceived research participation risk were associated with decreased willingness to participate in future health research and if health research knowledge was associated with increased willingness to participate among Chinese, Korean, South Asian, North American Latiné, South American Latiné, and SWANA NYC residents.

## Methods

This study used data collected via the Cancer Community Health Resources and Needs Assessment (Cancer CHRNA), a cross-sectional survey developed by NYU Langone Health Department of Population Health, NYU Langone Perlmutter Cancer Center, and 23 community-based organizations (CBO’s). All relevant survey questions can be found in Appendix Table 4. From October 2021-December 2022, written consent was obtained, and data was collected. Participants included 2,636 NYC residents aged 18 years and older who were fluent in one of the ten survey languages (Arabic, Bangla, Chinese [Simplified or Traditional], English, Haitian Creole, Korean, Russian, Spanish, or Urdu). Surveys were translated using translation services and were reviewed by bilingual staff and community partners. Discrepancies in translations were resolved through consensus. The questions were tested by staff in REDCap before survey administration, and edits were made as needed. The Cancer CHRNA leveraged CBO partners and used a multi-pronged convenience sampling approach to reach historically underrepresented groups who were intentionally oversampled to enable disaggregated data collection across ethnic subgroups. Additional information on Cancer CHRNA recruitment and survey administration methods has been previously reported [34]. The institutional IRB approved the study protocol, survey, and recruitment materials, and participants were consented before participation. Data was obtained from NYU Langone Health via a data use agreement.

### Measures

#### Outcome

*Willingness to participate in health research* was measured with the question “Would you ever participate in another health research study?” with answer choices Yes, No, Don’t know/Not sure, and Decline to state. This question came immediately after a question asking about timing of participation in cancer clinical trials and a question defining a survey as a type of health research study. Both questions could have impacted interpretation of and response to the outcome question.

#### Exposures

*Medical mistrust* was examined as a continuous variable and was measured via responses on a 5-point Likert scale (1 = Strongly Disagree, 5 = Strongly Agree) to nine statements (e.g., “The Health Care System does its best to make patients’ health better”) sourced from the Health Care System Distrust Scale [35] (see Appendix Table 5 for all statements). Responses to statements (a), (c), (f), and (g) were reverse coded such that higher scores indicate more mistrust. When responses to two or less statements were missing, the mean of the answered statements was imputed for the missing statements. The nine responses were summed, creating a continuous variable ranging from 9 (least mistrust) to 45 (most mistrust).

*Clinical trial knowledge *was measured with the question, “How knowledgeable would you say you are about clinical trials?” with response options of Very knowledgeable, Somewhat knowledgeable, Not very knowledgeable, and Not at all knowledgeable. Clinical trial knowledge was examined as a binary variable with categories Very/Somewhat knowledgeable and Not very/Not at all knowledgeable.

*Clinical trial risk *was measured with the question, “In your opinion, how much risk, if any, is associated with participating in clinical trials?” with answer choices of A lot of risk, Some risk, and No risk at all.

#### Effect measure modifier

Race and ethnicity were collected with the question: “*What is your race or ethnic background? (check all that apply)*” with response options of White; Hispanic, Latino, or Spanish origin; Black; Middle Eastern or North African; Native Hawaiian or Pacific Islander; Asian; American Indian, Native, First Nations, Indigenous Peoples of the Americas, or Alaska Native; Some other Race or Origin (please specify); Don’t Know/Not Sure; and Decline to state. For each racial category selected (besides Some other Race or Origin (please specify); Don’t Know/Not Sure; and Decline to state), a follow-up question was asked: “*Which group(s) best represents your origin or ancestry?*” with answer choices specific to each racial or ethnic group, Other (please specify), Don’t Know/Not Sure, and Decline to state. Language and birth country were used to recode missing ethnicities when applicable.

Using ethnic subgroup data, a mutually exclusive racial, ethnic, or regional identity variable was created. The categories included Chinese, Korean, South Asian (including Asian Indian, Bangladeshi, Nepali, and Pakistani individuals), Haitian, North American Latiné (including Costa Rican, Cuban, Dominican, Guatemalan, Honduran, Mexican, Nicaraguan, Panamanian, Puerto Rican, and Salvadoran individuals), South American Latiné (including Bolivian, Brazilian, Chilean, Colombian, Ecuadorian, Paraguayan, Peruvian, and Venezuelan individuals), and Middle Eastern or North African (now referred to as SWANA; including Afghan, Algerian, Egyptian, Iranian, Iraqi, Israeli, Jordanian, Lebanese, Moroccan, Palestinian, Saudi, Syrian, and Yemeni individuals). The split into North American Latiné and South American Latiné from the Latiné group was determined by geography, migration patterns, sample size, and a previous publication, which examined several North American Latiné groups (e.g., Central American, Dominican, Mexican) individually and South American Latiné collectively [36]. We examined all racial, ethnic, or regional groups possible given sample size constraints.

Aggregated groups for Asian (including all participants within the Chinese, Korean, and South Asian groups) and Latiné (including all participants within the North American Latiné and South American Latiné groups) were also examined for comparison of aggregated vs. disaggregated findings. With Haitian individuals making up the only Black subgroup in this study and SWANA populations being an aggregated group as is, aggregate comparison groups were not examined for either.

All individuals identifying as more than one broader racial group (e.g., White, Asian) were excluded due to small sample sizes. While there are groups of White individuals who are often excluded from health research, including rural White communities, the present study aimed to focus on racial and ethnic research participation disparities. Therefore, White individuals were excluded from the present study. See the Appendix for further details on the racial, ethnic, or regional identity group variable.

**Table 2:** Multinomial regression models evaluating the odds of not being willing to participate in health research among All Asian, Chinese, Korean, South Asian, Haitian, All Latiné, North American Latiné, South American Latiné, and SWANA study participants controlled for age, sex assigned at birth, education level, and nativity

	**All Asian**^a^ **N=1066**		**Chinese** **N=537**		**Korean** **N=243**		**South Asian**^b^ **N=286**		**Haitian** **N=149**		**All Latiné**^c^ **N=315**		**North American ** **Latiné**^d^ **N=220**		**South American ** **Latiné**^e^ **N=95**		**SWANA**^f^ **N=264**	
	**OR** **[95% CI]**	**P**	**OR** **[95% CI]**	**P ** **(adj P)**^g^	**OR** **[95% CI]**	**P ** **(adj P)**^g^	**OR** **[95% CI]**	**P ** **(adj P)**^g^	**OR** **[95% CI]**	**P ** **(adj P)**^g^	**OR** **[95% CI]**	**P**	**OR** **[95% CI]**	**P ** **(adj P)**^g^	**OR** **[95% CI]**	**P ** **(adj P)**^g^	**OR** **[95% CI]**	**P ** **(adj P)**^g^
*Medical mistrust (continuous)*	1.03 [1.00, 1.06]	0.06	**1.06** **[1.00, 1.12]**	**0.05** (0.33)	1.05 [0.98, 1.12]	0.16 (1.00)	0.98 [0.94, 1.04]	0.55 (1.00)	**0.87** **[0.81, 0.94]**	**<0.001** **(<0.01)**	**1.08** **[1.01, 1.15]**	**0.02**	1.07 [0.99, 1.16]	0.08 (0.54)	**1.15** **[1.02, 1.30]**	**0.03** **(0.20)**	0.94 [0.89, 1.00]	0.07 (0.46)
*Clinical trial knowledge*																		
Very/Somewhat knowledgeable (ref)	–	–	–	–	–	–	–	–	–	–	–	–	–	–	–	–	--	--
Not very/Not at all knowledgeable	0.77 [0.56, 1.06]	0.12	**0.55** **[0.34, 0.88]**	**0.01** **(0.09)**	1.27 [0.50, 3.24]	0.61 (1.00)	1.11 [0.60, 2.06]	0.74 (1.00)	**2.77** **[1.15, 6.65]**	**0.03** **(0.16)**	1.19 [0.63, 2.23]	0.59	1.45 [0.68, 3.10]	0.34 (1.00)	0.43 [0.11, 1.74]	0.24 (1.00)	0.61 [0.32, 1.17]	0.14 (0.98)
*Clinical trial risk*																		
No risk at all (ref)	–	–	–	–	–	–	–	–	–	–	–	–	–	–	–	–	--	--
Some risk	1.22 [0.65, 2.29]	0.54	2.31 [0.55, 9.75]	0.25 (1.00)	0.36 [0.09, 1.40]	0.14 (0.99)	2.00 [0.68, 5.82]	0.21 (1.00)	3.85 [0.67, 21.99]	0.13 (0.91)	0.61 [0.22, 1.67]	0.34	0.98 [0.24, 3.99]	0.97 (1.00)	**0.13** **[0.02, 0.82]**	**0.03** **(0.21)**	0.86 [0.23, 3.16]	0.82 (1.00)
A lot of risk	1.85 [0.90, 3.81]	0.09	2.22 [0.49, 10.14]	0.30 (1.00)	1.44 [0.29, 7.24]	0.66 (1.00)	2.85 [0.81, 10.06]	0.10(0.73)	2.31 [0.38, 13.82]	0.36 (1.00)	0.53 [0.15, 1.80]	0.31	0.86 [0.17, 4.41]	0.86(1.00)	0.16 [0.01, 2.21]	0.17 (1.00)	1.53 [0.34, 6.84]	0.58 (1.00)

^a^Includes Chinese, Korean, and South Asian individuals

^b^Includes Asian Indian, Bangladeshi, Nepali, and Pakistani individuals

^c^Includes North American Latiné and South American Latiné individuals

^d^Includes Costa Rican, Cuban, Dominican, Guatemalan, Honduran, Mexican, Nicaraguan, Panamanian, Puerto Rican, and Salvadoran individuals

^e^Includes Bolivian, Brazilian, Chilean, Colombian, Ecuadorian, Paraguayan, Peruvian, and Venezuelan individuals

^f^Southwest Asian and North African; includes Afghan, Algerian, Egyptian, Iranian, Iraqi, Israeli, Jordanian, Lebanese, Moroccan, Palestinian, Saudi, Syrian, and Yemeni individuals

^g^Adjusted p-value using Bonferroni adjustment at number of groups = 7

^h^Bold indicates statistical significance at 𝛼=0.05 (unadjusted p-value)

#### Covariates

Age (continuous), birth sex (male, female), education level (binary variable with categories of ≤ High school graduate vs. > High school [Some college/vocational/technical school or higher]), and nativity (binary variable with categories of Born in the U.S. vs. Not born in the U.S.) were examined as covariates.

### Analyses

The Cancer CHRNA dataset included *n* = 2,636 participants; *n* = 2,051 identified as Chinese, Korean, South Asian, Haitian, North American Latiné, South American Latiné, or SWANA. Of the *n* = 2,051, *n* = 26 were excluded due to identifying as another racial or ethnic group/subgroup. Another *n* = 231 participants were excluded due to missing one or more of the variables described above. The analytic sample was *n* = 1,794. Frequencies and percentages of all categorical variables and means and standard deviations for all continuous variables were calculated overall, for the two aggregate groups, and stratified by racial, ethnic, or regional group.

Chi-square tests for categorical variables and Kruskal-Wallis tests for continuous variables were used to compare characteristics across groups. Multinomial regression analysis was used to examine the associations between medical mistrust, clinical trial knowledge, and clinical trial risk and willingness to participate in health research (Yes (ref), No, Don’t know/Not sure) adjusting for age, sex, education, and nativity. Collinearity was assessed with correlation analysis, and English proficiency was excluded as a covariate due to collinearity with both nativity and education level. Effect measure modification of racial, ethnic, or regional identity on the association between each exposure (medical mistrust, clinical trial knowledge, clinical trial risk) and willingness to participate in health research was examined in three separate multinomial regression models (one for each exposure). When the interaction term was significant (*p* < 0.10), models were stratified by racial, ethnic, or regional identity. All exposures and covariates were included in the same model once stratified by racial, ethnic, or regional identity. An additional model for each aggregate group was also examined. All analyses were conducted in SAS Studio version 3.81. Interaction significance was assessed at 𝛼=0.1; main model significance was assessed at 𝛼=0.05. Unadjusted and Bonferroni adjusted p-values are shown for regression analyses.

## Results

The largest racial, ethnic, or regional group in the analytic sample was Chinese (29.9%) followed by South Asian (15.9%), SWANA (14.7%), Korean (13.5%), North American Latiné (12.2%), Haitian (8.3%), and South American Latiné (5.3%) (Table 1). Most of the sample was female (62.0%), had education beyond high school (57.1%), and were not born in the U.S. (79.1%). The mean age of the sample was 44.3 years. Close to half of participants (46.0%) were willing to participate in a future health research study, 27.5% were not willing to participate, and 26.5% were unsure of their willingness to participate. The mean medical mistrust score was 25.5 (SD = 5.4). Most participants reported being Not very/Not at all knowledgeable about clinical trials (66.4%) and perceiving Some clinical trial risk (76.3%).

All variables differed significantly by racial, ethnic, or regional group (*p* < 0.0001), and results among All Asian participants and All Latiné participants are shown for comparison with disaggregated groups (Table 1). Mean age ranged from 37.4 years among South Asian participants to 49.4 years among Chinese participants. A larger proportion of females participated across all racial groups with the largest proportion of females among North American Latiné individuals (75.5%) and the smallest proportion among SWANA individuals (55.3%). South Asian participants were most likely to have had some education beyond high school (74.8%), and Chinese participants were least likely (36.1%). Korean participants were most likely to have been born outside of the U.S. (86.4%); North American Latiné participants were least likely (50.0%). South Asian participants were most likely to be willing to participate in health research (58.7%); Chinese participants were least likely (35.8%). Medical mistrust scores ranged from 24.3 (least mistrust) among Chinese participants to 29.0 (most mistrust) among Haitian participants. South Asian and Haitian participants self-reported the most clinical trial knowledge: 45.1% and 45.0%, respectively, reported being Very/somewhat knowledgeable. Korean individuals were least likely to report being Very/somewhat knowledgeable (16.9%). Haitian participants were most likely to report A lot of risk associated with clinical trial participation (42.3%). Among the other groups, perception of A lot of risk ranged from 11.6% among South American Latiné individuals to 17.7% among North American Latiné individuals.

**Table 3:** Multinomial regression models evaluating the odds of not knowing or not being sure of willingness to participate in health research among All Asian, Chinese, Korean, South Asian, Haitian, All Latiné, North American Latiné, South American Latiné, and SWANA study participants controlled for age, sex assigned at birth, education level, and nativity

	**All Asian**^a^ **N=1066**		**Chinese** **N=537**		**Korean** **N=243**		**South Asian**^b^ **N=286**		**Haitian** **N=149**		**All Latiné**^c^ **N=315**		**North American ** **Latiné**^d^ **N=220**		**South American ** **Latiné**^e^ **N=95**		**SWANA**^f^ **N=264**	
	**OR** **[95% CI]**	**P**	**OR** **[95% CI]**	**P ** **(adj P)**^g^	**OR** **[95% CI]**	**P ** **(adj P)**^g^	**OR** **[95% CI]**	**P ** **(adj P)**^g^	**OR** **[95% CI]**	**P ** **(adj P)**^g^	**OR** **[95% CI]**	**P**	**OR** **[95% CI]**	**P ** **(adj P)**^g^	**OR** **[95% CI]**	**P ** **(adj P)**^g^	**OR** **[95% CI]**	**P ** **(adj P)**^g^
*Medical mistrust (continuous)*	**1.07** **[1.03, 1.10]**	**<0.0001**	1.05 [0.99, 1.11]	0.09 (0.63)	**1.13** **[1.05, 1.22]**	**<0.01** **(0.01)**	**1.06** **[1.01, 1.12]**	**0.02** **(0.15)**	**0.91** **[0.84, 0.99]**	**0.03** **(0.19)**	**1.10** **[1.04, 1.16]**	**<0.01**	**1.18** **[1.10, 1.28]**	**<0.0001** **(<0.001)**	0.98 [0.89, 1.08]	0.69 (1.00)	**0.91** **[0.86, 0.97]**	**<0.01** **(0.03)**
*Clinical trial knowledge*																		
Very/Somewhat knowledgeable (ref)	–	–	–	–	–	–	–	–	–	–	–	–	–	–	–	–	--	--
Not very/Not at all knowledgeable	1.36 [0.96, 1.94]	0.09	1.11 [0.66, 1.85]	0.69 (1.00)	1.06 [0.40, 2.83]	0.91 (1.00)	**2.09** **[1.07, 4.07]**	**0.03** **(0.22)**	2.68 [0.98, 7.34]	0.06 (0.39)	1.70 [0.98, 2.95]	0.06	1.76 [0.88. 3.52]	0.11 (0.76)	2.35 [0.80, 6.95]	0.12 (0.85)	**2.71** **[1.21, 6.03]**	**0.02** **(0.11)**
*Clinical trial risk*																		
No risk at all (ref)	–	–	–	–	–	–	–	–	–	–	–	–	–	–	–	–	--	--
Some risk	1.19 [0.62, 2.28]	0.61	1.41 [0.38, 5.20]	0.61 (1.00)	2.62 [0.27, 25.69]	0.41 (1.00)	0.80 [0.32, 1.98]	0.63 (1.00)	7.21 [0.64, 81.16]	0.11 (0.77)	1.66 [0.56, 4.95]	0.37	0.99 [0.27, 3.69]	0.99 (1.00)	3.39 [0.35, 33.13]	0.29 (1.00)	0.39 [0.13, 1.20]	0.10 (0.71)
A lot of risk	1.58 [0.75, 3.33]	0.23	1.59 [0.40, 6.37]	0.51 (1.00)	5.33 [0.44, 64.29]	0.19 (1.00)	0.89 [0.28, 2.81]	0.84 (1.00)	5.47 [0.48, 63.05]	0.17 (1.00)	1.30 [0.38, 4.47]	0.68	1.02 [0.24, 4.40]	0.98 (1.00)	1.05 [0.07, 16.41]	0.97 (1.00)	0.34 [0.08, 1.55]	0.16 (1.00)

^a^Includes Chinese, Korean, and South Asian individuals

^b^Includes Asian Indian, Bangladeshi, Nepali, and Pakistani individuals

^c^Includes North American Latiné and South American Latiné individuals

^d^Includes Costa Rican, Cuban, Dominican, Guatemalan, Honduran, Mexican, Nicaraguan, Panamanian, Puerto Rican, and Salvadoran individuals

^e^Includes Bolivian, Brazilian, Chilean, Colombian, Ecuadorian, Paraguayan, Peruvian, and Venezuelan individuals

^f^Southwest Asian and North African; includes Afghan, Algerian, Egyptian, Iranian, Iraqi, Israeli, Jordanian, Lebanese, Moroccan, Palestinian, Saudi, Syrian, and Yemeni individuals

^g^Adjusted p-value using Bonferroni adjustment at number of groups = 7

^h^Bold indicates statistical significance at 𝛼=0.05 (unadjusted p-value)

Evidence of effect measure modification was found for racial, ethnic, or regional identity on the relationship between medical mistrust (*p* < 0.0001), clinical trial knowledge (*p* = 0.03), and clinical trial risk (*p* = 0.03) and willingness to participate in health research (data not shown). Tables 2 and 3 present findings from adjusted multinomial models examining the association between these three exposures and willingness to participate in health research (not willing or not knowing/being unsure of willingness vs. willing to participate), stratified by racial, ethnic, or regional identity. Overall F-tests (not shown)—which examine associations between the exposure and outcome across all levels of the outcome combined—indicated significant associations between medical mistrust and willingness to participate for all groups except South American Latiné (*p* = 0.06) and Chinese (*p* = 0.10) individuals. Associations of clinical trial knowledge with willingness to participate were significant for Chinese (*p* < 0.01), All Asian (*p* < 0.01), Haitian (*p* = 0.05), and SWANA (*p* < 0.01) participants. Trial risk associations were only significant overall for Korean individuals (*p* = 0.03); however, no associations were observed among Korean individuals when examining the associations for each outcome level comparison.

Table 2 presents the fully adjusted multinomial model outputs for the outcome of *not* being willing to participate in health research vs. being willing to participate in health research stratified by racial, ethnic, or regional identity. While overall F-tests were not significant for medical mistrust and participation willingness among Chinese and South American Latiné individuals, Table 2 shows significant associations when looking at the specific outcome comparison of *not* being willing to participate vs. being willing to participate. As medical mistrust increased, the odds of *not* being willing to participate vs. being willing to participate increased among Chinese (OR: 1.06, 95%CI: 1.00, 1.12) and South American Latiné (OR: 1.15, 95%CI: 1.02, 1.30) participants. Conversely, among Haitian participants, as medical mistrust increased, the odds of *not* being willing to participate vs. being willing to participate *decreased* (OR: 0.87, 95%CI: 0.81, 0.94). Finally, among All Latiné participants, a positive association was found between increased medical mistrust and willingness to participate (OR: 1.08, 95%CI: 1.01, 1.15)–as among South American Latiné participants–despite no findings of such a relationship among Noth American Latiné participants.

Haitian participants who reported being Not very/Not at all knowledgeable compared to being Somewhat/Very knowledgeable about clinical trials had 2.77 (95%CI: 1.15, 6.65) times higher odds of *not* being willing to participate vs. being willing to participate. Conversely, Chinese participants who reported being Not very/Not at all knowledgeable about clinical trials compared to being Somewhat/Very knowledgeable had 0.55 (95%CI: 0.34, 0.88) times lower odds of *not* being willing to participate in health research vs. being willing to participate. South American Latiné individuals who reported perceiving Some risk compared to No risk associated with health research participation had 0.13 (95%CI: 0.02, 0.82) times *lower* odds of *not* being willing to participate in health research vs. being willing to participate (overall F-test: *p* = 0.07).

Table 3 presents the fully adjusted multinomial model outputs for the outcome of *not knowing/being unsure* of willingness to participate in health research vs. being willing to participate in health research stratified by racial, ethnic, or regional identity. As medical mistrust increased, the odds of not knowing/being unsure of willingness to participate vs. being willing to participate increased among Korean (OR: 1.13, 95%CI: 1.05, 1.22), South Asian (OR: 1.06, 95%CI: 1.01, 1.12), and North American Latiné (OR: 1.18, 95%CI: 1.10, 1.28) participants. The same was found when aggregating among All Asian participants (OR: 1.07, 95%CI: 1.03, 1.10) and All Latiné participants (OR: 1.10, 95%CI: 1.04, 1.16). Conversely, as medical mistrust increased, the odds of not knowing/being unsure of willingness to participate vs. being willing to participate *decreased* among Haitian (OR: 0.91, 95%CI: 0.84, 0.99) and SWANA (OR: 0.91, 95%CI: 0.86, 0.97) participants. South Asian (overall F-test: *p* = 0.10) and SWANA participants who reported being Not very/Not at all knowledgeable of clinical trials compared to being Somewhat/Very knowledgeable of clinical trials had higher odds of not knowing/being unsure of willingness to participate vs. being willing to participate (OR: 2.09, 95%CI: 1.07, 4.07; OR: 2.71, 95%CI: 1.21, 6.03, respectively).

## Discussion

In this study across seven NYC racial, ethnic, or regional groups, nearly half of the analytic sample expressed willingness to participate in future health research studies, with variation across groups. This level of willingness aligns with the levels among the general U.S. population,^37^ challenging assumptions around minoritized populations not being willing to participate in research. Levels of medical mistrust, clinical trial knowledge, and perceived clinical trial risk and their association with willingness to participate varied by group, indicating heterogeneity in factors influencing research participation across diverse groups in NYC. Further, associations among aggregated All Asian and All Latiné groups differed from the associations among the disaggregated subgroups (Chinese, Korean, and South Asian; and North American Latiné and South American Latiné, respectively), indicating the importance of data disaggregation to accurately examine diverse groups.

Although we examined willingness to *not* participate, interpreting our findings within the context of existing literature is more easily understood when framed as *less willingness* to participate. We found that increased medical mistrust was associated with less willingness to participate in future health studies among Chinese and South American Latiné participants; among Haitian participants, increased mistrust was associated with *increased* willingness to participate. Among Korean, South Asian, and North American Latiné participants, more mistrust was associated with higher odds of *not knowing* their willingness to participate (vs. being willing). Conversely, more mistrust was associated with *lower* odds of not knowing their willingness to participate among Haitian and SWANA participants.

Chinese participants reporting less clinical trial knowledge were *more* likely to be willing to participate in future research studies, while Haitian participants reporting less clinical trial knowledge were less likely to be willing. South Asian and SWANA participants reporting less clinical trial knowledge had higher odds of not knowing their willingness. South American Latiné participants reporting Some clinical trial risk (vs. No risk at all) were *more* likely to be willing to participate in future health research.

To our knowledge, this study is the first to examine the associations of medical mistrust, clinical trial knowledge, and perceived clinical trial risk with research participation willingness among the included NYC groups. Our findings indicate the need for tailored research recruitment interventions—including collaboration with CBOs and community health workers (CHWs)—and are important for guiding efforts to increase research participation across diverse groups.

### Willingness to participate

Our findings align previous studies indicating differences in willingness to participate across diverse groups [38, 39], including Liu et al.’s 2019 study among *n* = 17,339 Asian, Black, Latiné, Caucasian, and “other minority” U.S. residents [39]. While our findings on differences in participation willingness across ethnic groups align, Liu et al. consistently found higher rates of participation willingness among their racial and ethnic groups compared the subgroups examined in our study (i.e., Liu’s Asian American group had a higher rate than Chinese participants of this study—though similar rates were found among Korean, South Asian, and All Asian participants on aggregate, the Black American group had a higher rate than Haitian participants, and Liu’s Latiné participants had higher rates than this study's North and South American Latiné participants) [39]. 

Our lower rates may stem from nearly 80% of our sample having immigrated to the U.S., with just over half speaking English well or very well. Immigrants face unique challenges to research participation, including limited access to insurance and health information, and concerns about legal consequences [40]. Moreover, underrepresentation of immigrant and LEP populations in health and research professions may prevent immigrant, migrant, and LEP groups from using lived experiences to inform inclusive recruitment and retention practices [40]. 

### Medical mistrust

Findings on the association between medical mistrust and willingness to participate varied by group. Findings among Chinese, Korean, South Asian, and North and South American Latiné participants aligned with previous findings among Chinese, Asian, and Latiné individuals in the U.S., which indicate mistrust is inversely associated with participation willingness [11]. However, increased mistrust was positively associated with participation willingness among Haitian and SWANA participants, a finding both counterintuitive and missing from the literature among Black and Asian American individuals. A CHW who collected data for the Cancer CHRNA indicated that this finding may be partially explained by health literacy, which was not captured in the Cancer CHRNA survey. Health literacy could be linked to both more awareness of historical mistreatment of minoritized groups, leading to more medical mistrust, and more awareness of the benefits of clinical research and current protections for research participants, leading to more willingness to participate.

Existing literature among racial and ethnic groups on the association between health literacy and (a) medical mistrust and (b) willingness to participate is varied [41]. Tsai et al.’s 2018 study among Taiwanese individuals [42] indicates a positive relationship between trust and health literacy (examined with focus on providing and understanding health information), while White et al.’s 2014 study among Latiné individuals (using the Short Test of Functional Health Literacy in Adults) indicates an inverse relationship [43]. A 2020 review of barriers to health literacy among African Americans found medical mistrust to be a barrier to health literacy,^44^ yet, a 2015 study among Haitian American immigrants found no significant difference in health literacy (using the Brief Health Literacy Screen) between those with little to no trust in doctors compared to those with some or a lot of trust [45]. Considering the variation in findings and lack of research among Haitian and SWANA U.S. residents, additional qualitative research—conducted with input from CBOs and CHWs—into the relationships between health literacy, medical mistrust, and willingness to participate among these groups is needed.

### Clinical trial knowledge

Among Haitian, South Asian, and SWANA participants, less clinical trial knowledge was associated with less or unknown willingness to participate in health research, aligning with previous findings among African American and Asian American individuals [11]. However, among Chinese participants, *less* knowledge was associated with *more* willingness to participate. Several factors could be responsible, including trust in healthcare providers mediating the relationship between less knowledge and willingness to participate. A 2014 study among *n* = 3,159 Chicago-area Chinese adults aged ≥ 60 years found that participants had high levels of trust in physicians, and that being female, being born in China, and having lower education was associated with this increased physician trust.^46^ Given that these participants’ characteristics reflect the majority of Chinese participants in our study, and that lower education and less clinical trial knowledge are correlated, it is possible that elevated healthcare provider trust underpins the observed associations. Findings from a focus group study among *n* = 103 Asian Americans in Philadelphia, most of whom were of Chinese descent, found doctor recommendations to be a motivator for clinical trial participation and lack of trust in healthcare providers to be a barrier.^47^ These findings, though sparse, indicate the potential for mediation; however, they should be followed up with further studies examining these relationships among Chinese individuals.

### Clinical trial risk

Among South American Latiné participants, Some perceived clinical trial risk—compared to No perceived risk—was associated with more willingness to participate in health research. This counterintuitive finding may be due to > 77% of South American Latiné participants indicating Some perceived clinical trial risk, resulting in small sample sizes for the other risk categories. Some risk was chosen by the majority of participants across racial and ethnic groups, and this may contribute to the lack of findings overall between perceived risk and participation willingness.

### Strengths and limitations

This study has several major strengths, including collecting detailed racial-, ethnic-, and national-level data—allowing for disaggregated analyses across ethnic, national, and regional groups—along with extensive collaboration with a large, multilingual CBO and CHW team to recruit for and administer the survey, which created novel opportunities to engage historically underrepresented groups and LEP individuals in research. However, other historically underrepresented groups, including Native American and Multiracial/Multiethnic individuals, could not be included in analyses due to small sample sizes. Another limitation is the study population being a convenience sample, limiting generalizability. Further, discrepancies in overall F-test results vs. outcome level-specific findings (e.g., significant findings of the association between increased perceived clinical trial risk and more willingness to participate among South American Latiné individuals despite non-significant findings on the overall F test) may be attributed to small sample sizes. These discrepancies indicate the need for future research to confirm findings reported in this study. Finally, collecting perceived clinical trial risk as a three-category variable likely led to most participants selecting the middle category (“some risk”) and contributed to the lack of findings between risk and willingness to participate.

## Conclusions

This study is the first, to our knowledge, to examine the associations of medical mistrust, clinical trial knowledge, and perceived clinical trial risk with willingness to participate in health research among Chinese, Korean, South Asian, Haitian, North American Latiné, South American Latiné, and SWANA individuals in the U.S. The varied findings across groups in the presence, direction, and strength of associations between medical mistrust, clinical trial knowledge, and perceived clinical trial risk with willingness to participate—including between disaggregated and aggregated groups—along with the lack of U.S. research participation among diverse individuals, indicate a need for more racially and ethnically disaggregated research into factors impacting participation. This additionally indicates the need for tailored interventions to improve recruitment and retention of these groups in health research. Large portions of each study group reported willingness to participate in health research, indicating that researchers can find more diverse study populations. However, willingness to participate does not imply trust in the research process. It is imperative that researchers work with CBOs and CHWs from the communities that studies aim to reach in order to ensure culturally appropriate and linguistically tailored recruitment. This will demonstrate representation of target participant groups within the medical research system, bolstering accessibility of research participation and building participant trust with the study team and the health research process overall.

## Data Availability

Data generated in this study are not publicly available due to information that could compromise patient privacy/consent but are available upon reasonable request from the corresponding author.

## Appendix

**Table 4:** Relevant Survey Questions

**Question/Statement**	**Response Options**
What is your race or ethnic background? (check all that apply)	· White · Hispanic, Latino, or Spanish origin · Black · Middle Eastern or North African · Native Hawaiian or Pacific Islander · Asian · American Indian, Native, First Nations, Indigenous Peoples of the Americas, or Alaska Native · Some other Race or Origin (please specify): _________________________________________ · Don’t Know/Not Sure · Decline to state
Were you born in the United States?	· Yes · No · Don’t Know/Not Sure
What is the highest grade or level of schooling you completed?	· Less than 8 years · 8 through 11 years · 12 years or completed high school · Post high school training other than college (vocational or technical) · Some college · College graduate · Postgraduate
What was your sex assigned at birth?	· Male · Female · Don’t Know/Not Sure · Prefer not to answer
What is your age?	Write in response
How well do you speak English?	· Very well · Well · Not well · Not at all · Don’t Know/Not Sure
How knowledgeable would you say you are about clinical trials?	· Very knowledgeable · Somewhat knowledgeable · Not very knowledgeable · Not at all knowledgeable
In your opinion, how much risk, if any is associated with participating in clinical trials?	· A lot of risk · Some risk · No risk at all
When during a cancer journey do you believe it is best to enroll in a clinical trial? (Check all that apply):	· Immediately after diagnosis · In combination with the standard course of therapy · After all other treatment options have been exhausted · Never *– Selecting this option will clear your previous selections for this checkbox field* · Other (please specify):________________________________
Before today, have you ever participated in a health research study? For instance, this survey is a health research study.	· Yes · No, this is my first · Don’t Know/Not Sure · Decline to State
Would you ever participate in another health research study?	· Yes · No · Don’t Know/Not Sure · Decline to State

**Table 5:** Medical Mistrust Statements and Response Options

**Statement**	**Response Options**
(a) The Health Care System does its best to make patients’ health better	1. Strongly disagree 2. Disagree 3. Neither agree nor disagree 4. Agree 5. Strongly agree
(b) The Health Care System covers up its mistakes	1. Strongly disagree 2. Disagree 3. Neither agree nor disagree 4. Agree 5. Strongly agree
(c) Patients receive high quality medical care from the Health Care System	1. Strongly disagree 2. Disagree 3. Neither agree nor disagree 4. Agree 5. Strongly agree
(d) The Health Care System makes too many mistakes	1. Strongly disagree 2. Disagree 3. Neither agree nor disagree 4. Agree 5. Strongly agree
(e) The Health Care System puts making money above patients’ needs	1. Strongly disagree 2. Disagree 3. Neither agree nor disagree 4. Agree 5. Strongly agree
(f) The Health Care System gives excellent medical care	1. Strongly disagree 2. Disagree 3. Neither agree nor disagree 4. Agree 5. Strongly agree
(g) Patients get the same medical treatment from the Health Care System, no matter what the patient’s race or ethnicity	1. Strongly disagree 2. Disagree 3. Neither agree nor disagree 4. Agree 5. Strongly agree
(h) The Health Care System lies to make money	1. Strongly disagree 2. Disagree 3. Neither agree nor disagree 4. Agree 5. Strongly agree
(i) The Health Care System experiments on patients without them knowing.	1. Strongly disagree 2. Disagree 3. Neither agree nor disagree 4. Agree 5. Strongly agree

### Description of effect measure modifier: racial, ethnic, or regional subgroup variable

Race and ethnicity were collected with the question: “*What is your race or ethnic background? (check all that apply)*” with response options of White; Hispanic, Latino, or Spanish origin; Black; Middle Eastern or North African; Native Hawaiian or Pacific Islander; Asian; American Indian, Native, First Nations, Indigenous Peoples of the Americas, or Alaska Native; Some other Race or Origin (please specify); Don’t Know/Not Sure; and Decline to state. For each racial category selected (besides Some other Race or Origin (please specify); Don’t Know/Not Sure; Decline to state), a follow-up question was asked: “*Which group(s) best represents your origin or ancestry?*” with answer choices specific to each racial group, Other (please specify), Don’t Know/Not Sure, and Decline to state. Language and country of birth were used to recode missing ethnicities when applicable.

For those who selected “Asian,” the ethnic group response options were Chinese, Asian Indian, Filipino, Korean, Japanese, Vietnamese, Guyanese, Bangladeshi, and Pakistani. For those who selected “Black,” the ethnic group response options were African American, Jamaican, Guyanese, Haitian, Trinidadian and Tobagonian, Nigerian, Ghanaian, Ethiopian, and Somali. For those who selected “Latiné,” the ethnic group response options were Puerto Rican, Dominican, Mexican, Colombian, and Ecuadorian. Detailed ethnic groups were asked for those choosing Middle Eastern or North African, but the final sample was not large enough to disaggregate these groups. Country of birth, self-reported language spoken at home, and survey completion language were used to impute race and ethnicity when these questions were skipped. To place participants into appropriate groupings, some write-in responses were recategorized (e.g., White other: ‘Syrian’ was moved to SWANA: Syrian). All racial and ethnic groups were coded as binary variables (1=individual belongs to group; 0=individuals does not self-identify as belonging to group).

The Asian ethnic subgroup variables were used to create three mutually exclusive Asian ethnic subgroups: Chinese, Korean, and South Asian (including Asian Indian, Bangladeshi, Nepali, and Pakistani). Of the Black subgroups, only Haitian individuals were included in analysis due to sample size constraints. The Latiné ethnic group was separated into a mutually exclusive subgroup variable with categories of: North American Latiné (including Costa Rican, Cuban, Dominican, Guatemalan, Honduran, Mexican, Nicaraguan, Panamanian, Puerto Rican, and Salvadoran individuals) and South American Latiné (including Bolivian, Brazilian, Chilean, Colombian, Ecuadorian, Paraguayan, Peruvian, and Venezuelan individuals). The split into North American Latiné and South American Latiné from the Latiné group was determined by geography, migration patterns, sample size, a previous publication, which examined several North American Latiné groups (e.g., Central American, Dominican, Mexican) individually and South American Latiné collectively.^24^ All North American Latiné subgroups were combined due to small sample sizes. Participants included in the mutually exclusive Asian and Latiné ethnic subgroup variables or identifying as Haitian or as Middle Eastern/North African (via the first race and ethnicity question) were then coded into a mutually exclusive racial, ethnic, or regional identity variable with the categories Chinese, Korean, South Asian, Haitian, North American Latiné, South American Latiné, and Middle Eastern or North African (now referred to as Southwest Asian or North African [SWANA], which includes Afghan, Algerian, Egyptian, Iranian, Iraqi, Israeli, Jordanian, Lebanese, Moroccan, Palestinian, Saudi, Syrian, and Yemeni individuals). All participants included in this variable were then examined for identification with any other racial group (i.e., as measured via the first race and ethnicity question). All individuals identifying as more than one racial group were excluded due to small sample sizes.

Aggregated groups for Asian (including all participants within the Chinese, Korean, and South Asian groups) and Latiné (including all participants within the North American Latiné and South American Latiné groups) were also examined for comparison of aggregated vs. disaggregated findings. With Haitian individuals making up the only Black subgroup in this study and SWANA populations being an aggregated group as is, aggregate comparison groups were not examined for either.
